# Treatment of a severe class II subdivision malocclusion following failed bimaxillary anterior segment osteotomy: a case report

**DOI:** 10.1186/s12903-025-06000-7

**Published:** 2025-04-21

**Authors:** Viet Anh Nguyen, Thi Minh Anh Ha

**Affiliations:** 1 Faculty of Dentistry, PHENIKAA University, Yen Nghia, Ha Dong, Hanoi, 12116 Vietnam; 2 Private Practice, Viet Anh Orthodontic Clinic, Hanoi, Vietnam

**Keywords:** Angle class II malocclusions, Facial asymmetry, Orthodontic anchorage techniques, Orthodontic appliances, Orthognathic surgical procedures

## Abstract

**Background:**

Orthognathic surgery without a well-coordinated orthodontic plan can compromise aesthetic and functional outcomes, potentially necessitating complex orthodontic retreatment.

**Case presentation:**

This case report presents a 28-year-old female with residual extraction spaces and upper incisor proclination following a failed bimaxillary anterior segment osteotomy. Clinical examination revealed a convex profile, Class I skeletal base relationship, hyperdivergent facial pattern, full-cusp Class II subdivision on the left side, excessive overjet, and significant lower dental midline deviation. Treatment involved digitally planned straight-wire lingual appliances combined with miniscrew anchorage to distalize the entire upper arch and lower right quadrant, while mesializing the lower left quadrant. This approach successfully corrected the malocclusion, achieving a solid Class I relationship, normal overbite and overjet, and a harmonious profile.

**Conclusions:**

This case highlights the effective management of complex malocclusions arising from suboptimal orthognathic surgery through a combination of miniscrew-assisted distalization and mesialization techniques, aided by digital planning and lingual appliances.

## Introduction

Orthognathic surgery is typically performed to correct severe skeletal discrepancies that cannot be resolved with orthodontic treatment alone. However, achieving successful surgical outcomes depends heavily on a comprehensive orthodontic treatment plan. Without a well-coordinated plan, aesthetic and functional results could be compromised, potentially leading to suboptimal outcomes [1]. Unsatisfactory outcomes can lead patients to seek further treatment, such as orthodontic retreatment for mild to moderate discrepancies or, in severe cases, a second orthognathic surgery [2].

Class II subdivision malocclusion is a multifactorial condition with a complex etiology. Whereas hereditary components, childhood propensities, and injury are frequently relevant, the correct cause can be challenging to pinpoint. Some studies suggest that mandibular body asymmetry plays a noteworthy part, whereas others highlight the significance of condylar asymmetry [3–5]. Furthermore, dental variables such as unilateral mesial positions of upper molars and distal positions of lower molars may contribute to the asymmetric anteroposterior relationship. Additionally, distal movements of premolars and canine following premature unilateral mandibular molar missing can lead to a Class II subdivision. A large overjet and lower dental midline deviation to the Class II side may become a major concern, promoting patients seeking orthodontic treatment.

Management of Class II subdivision malocclusion is generally challenging due to the need for asymmetric force and anchorage systems, frequently including skeletal anchorage [6, 7]. Adult patients often prioritize aesthetics during orthodontic treatment, leading to the popularity of invisible options such as lingual braces and clear aligners. However, for complex cases requiring lower molar mesialization such as full-cusp Class II subdivisions, fixed appliances are generally preferred [8]. This preference stems from their easier integration with miniscrews, which are often necessary for the effective treatment of these challenging malocclusions [9].

This case report presents the invisible orthodontic treatment of an adult patient with a Class II subdivision malocclusion with straight-wire lingual appliances and miniscrews. The patient had previously undergone orthognathic surgery, which unfortunately did not yield satisfactory results. Consequently, the current treatment focuses on addressing an unresolved full-cusp Class II relationship on the left side, a lower midline deviation, and a large overjet.

## Case presentation

### Diagnosis and etiology

A 28-year-old female patient presented with chief complaints of residual extraction spaces and upper incisor proclination following orthognathic surgery. Her medical, family, and psychosocial histories were unremarkable and did not contribute to her current dental condition.

The patient reported that she had previously undergone a bimaxillary anterior segment osteotomy with the extraction of all first premolars to setback the anterior segment of both jaws and address her bimaxillary protrusion. This procedure was performed by a plastic surgeon without consulting an orthodontist, despite the presence of a distal occlusion on the left side and a mandibular dental midline deviation to the left. Unfortunately, the surgery did not yield satisfactory results in terms of completely closing the extraction spaces, achieving stable occlusion, and correcting the midline.

**Fig. 1:**
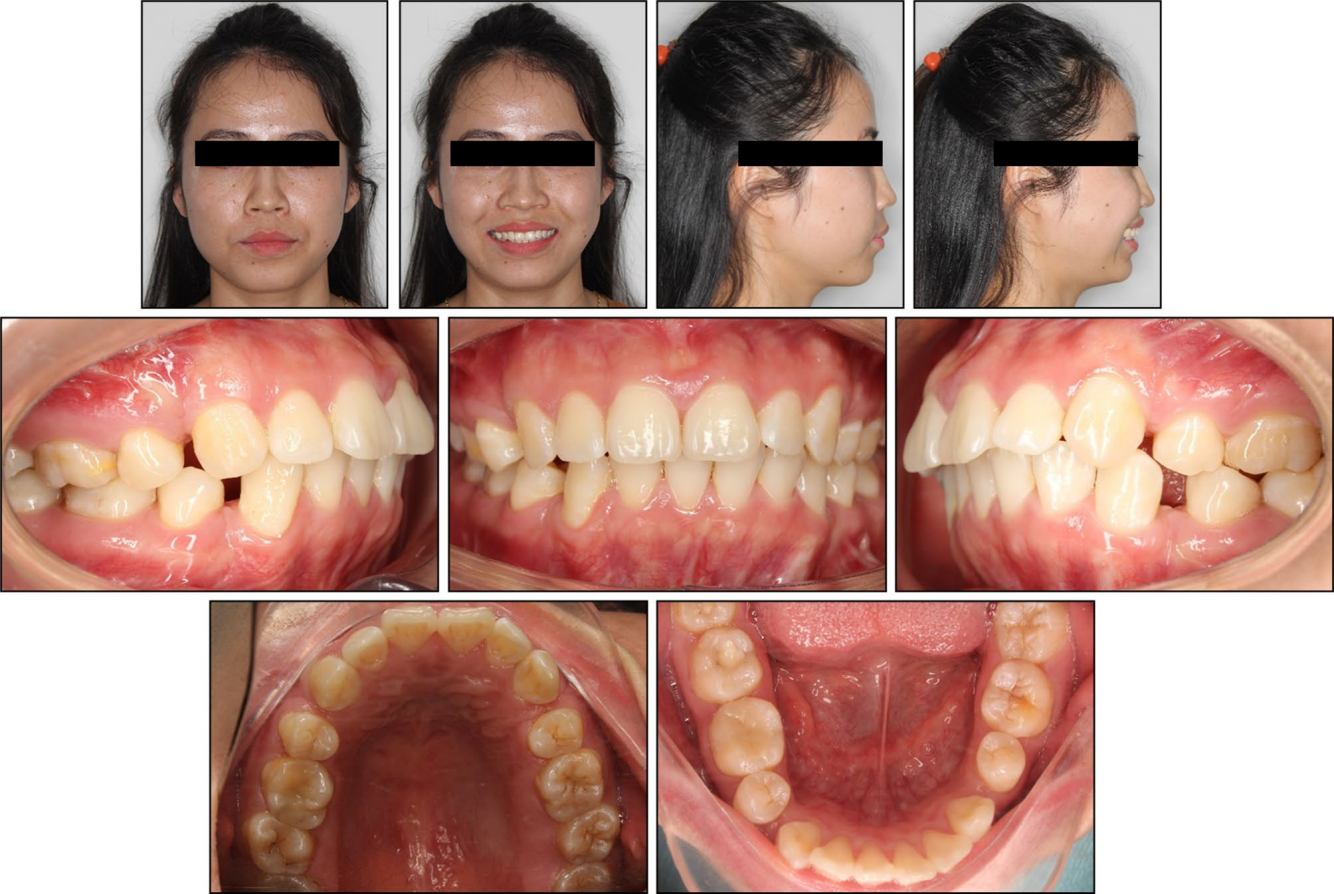
Pre-treatment extraoral and intraoral photographs

An extraoral frontal examination revealed a slightly elongated lower anterior facial third and facial asymmetry, with the mandible deviated to the left. A lateral examination indicated a convex facial profile with protrusive lips, accompanied by moderate muscular tension in the perioral and mentalis muscles on lip closure (Fig. 1). There were no signs indicating any disorder of the temporomandibular joint.

Intraoral examination showed a full-cusp Class II molar and canine relationship on the left side, but a Class I relationship on the right. The etiology of the Class II subdivision was supposed to be the premature loss of the lower left first molars. The maxillary midline coincided with the facial midline, while the mandibular midline deviated 5 mm to the left. Overjet and overbite measured 5 mm and 4 mm, respectively. The lower arch showed a deep curve of Spee. The lower right canine exhibited marked distolingual rotation. Residual spaces were noted in the maxillary and mandibular first premolar extraction sites, resulting from previous bimaxillary anterior segment osteotomy.

**Fig. 2:**
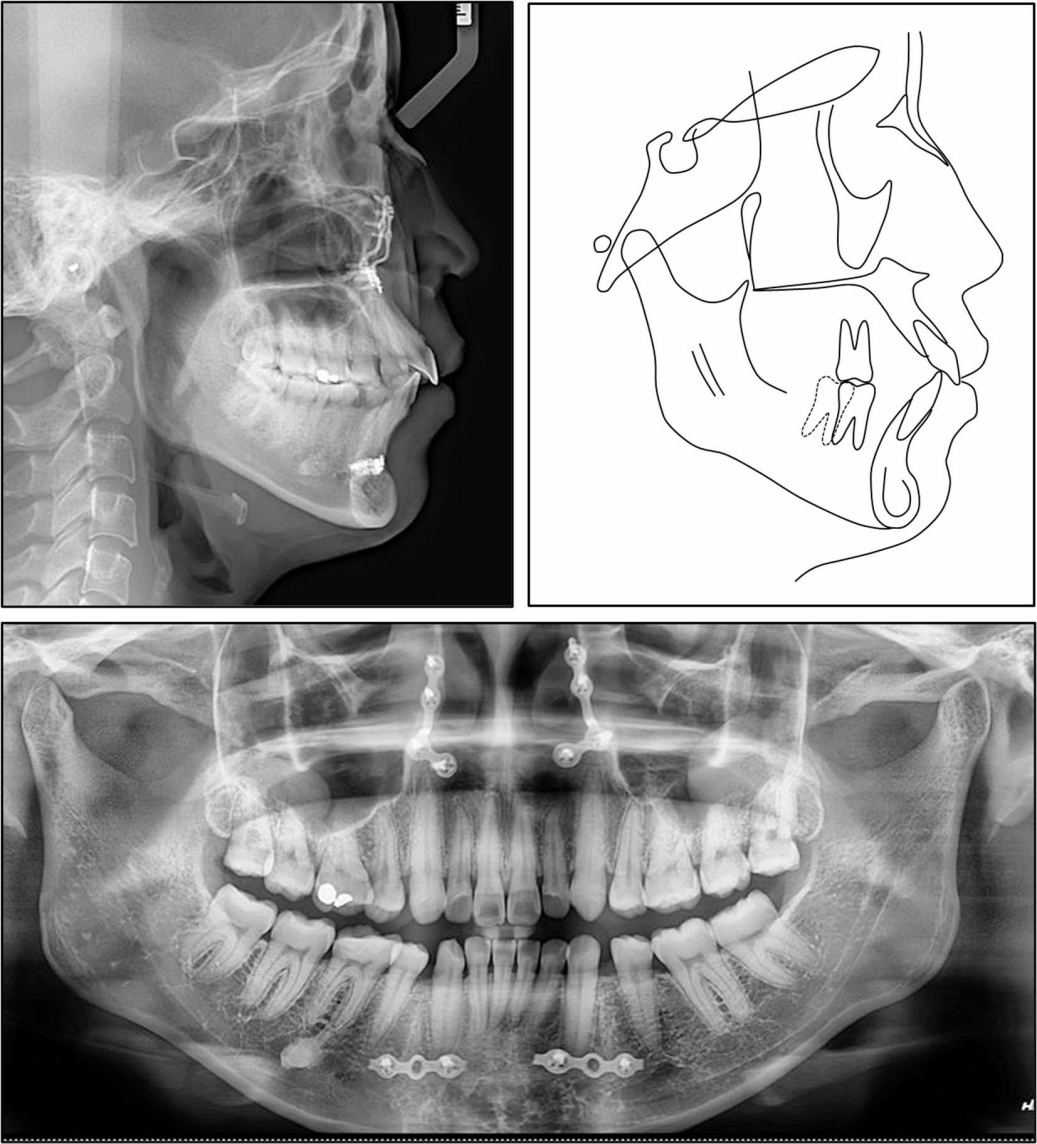
Pre-treatment cephalometric and panoramic radiographs and tracing

Cephalometric analysis revealed a Class I skeletal base relationship with Class II tendency characterized by a normal point A-nasion-point B angle (SNA) of 3.61°, with a well-positioned maxilla and mandible (Table 1). However, the patient exhibited a hyperdivergent facial pattern, as evidenced by an increased Frankfort-mandibular plane angle (FMA) of 31.37°. Dental analysis revealed labial inclinations of the upper and lower incisors, with an upper incisor to sella nasion (U1-SN) angle of 107.92°, and a lower incisor mandibular plane (L1-MP) angle of 96.91°. Soft tissue analysis demonstrated a slightly protrusive soft tissue profile, with the upper and lower lips protruding 1.56 mm and 2.02 mm beyond the E-line, respectively. The panoramic radiograph confirmed the absence of the lower left first molar and all four first premolars. The lower left incisors, canine, and second premolar exhibited distal tipping, while the lower left second and third molars tipped mesially, closing the space left by the missing first molar (Fig. 2).

**Table 1:** Cephalometric measurements

	Pretreatment	Posttreatment
Skeletal		
SNA (°)	79.19	79.07
SNB (°)	75.58	75.66
ANB (°)	3.61	3.10
FMA (°)	31.37	30.66
Dental		
U1-SN (°)	107.92	98.85
U1-NA (°)	28.73	19.79
U1-NA (mm)	8.3	4.24
L1-MP (°)	96.91	94.07
L1-NB (°)	30.51	27.40
L1-NB (mm)	8.76	6.65
Interincisal angle (°)	117.15	129.41
Soft tissue		
Upper lip/E-line (mm)	1.56	0.01
Lower lip/E-line (mm)	2.02	0.51

SNA = sella nasion point A; SNB = sella nasion point B; ANB = point A nasion point B; FMA = Frankfort mandibular plane angle; U1 = upper central incisor; L1 = lower central incisor; NB = nasion point B; NA = nasion point A; MP = mandibular plane

The hyperdivergent facial pattern (increased FMA) suggested avoiding further mandibular plane opening. The skeletal Class II relationship (increased ANB) highlighted the need for maxillary incisor retraction for bone remodeling at point A. The labial inclination of the incisors and the protrusive soft tissue profile reinforced the necessity for incisor retraction

### Treatment objectives

The primary goals of treatment were to address the patient’s chief complaints of remaining first premolar extraction spaces and a large overjet. In developing the treatment objectives, the cephalometric analysis was crucial in understanding the skeletal and dental discrepancies. The hyperdivergent facial pattern, indicated by the increased FMA, suggested that no further opening of the mandibular plane should be allowed. The skeletal Class II relationship, indicated by the increased ANB angle, highlighted the need for retraction of the maxillary incisors to achieve bone remodeling at point A. Additionally, the labial inclination of the upper and lower incisors, along with the protrusive soft tissue profile, further emphasized the necessity for incisor retraction. Therefore, the specific treatment objectives included (1) aligning the teeth and leveling the occlusal plane, (2) correcting the Class II molar and canine relationship on the left side, (3) closing existing extraction spaces, (4) obtaining an ideal overjet and overbite, (5) reducing the labial inclination of the upper and lower incisors, (6) aligning the lower dental midline with the upper dental and facial midline, and (7) maintaining or reducing the lower anterior facial height.

### Treatment alternatives

Given the patient’s history of orthognathic surgery and the existing Class I skeletal base relationship, a surgical treatment option was deemed unnecessary and therefore excluded. The patient’s treatment plan involved considering three potential options. The first option was a plan without extraction of any teeth including third molars. This plan aimed to create space for an implant at the site of the missing lower left first molar. This would be achieved by simultaneously distalizing the lower left second and third molars and mesializing the lower left second premolar, canine, and incisors. However, this approach was deemed less desirable for several reasons. Firstly, the patient’s facial convexity could have worsened due to the mesialization of the lower left teeth, which would increase protrusion. Secondly, the additional cost of an implant and crown was deemed unnecessary, given the patient’s concern with dental protrusion. As such, this option was excluded, as it would not align well with the patient’s esthetic priorities of reducing protrusion and improving facial balance.

The second alternative, also a non-extraction approach, involved mesializing the entire lower left quadrant to achieve a Class I dental relationship without the need for an implant. This would procline the lower incisors to obtain a normal overjet. While this approach would address the Class II malocclusion, it would not effectively reduce the patient’s dental protrusion. Moreover, the excessive mesialization required would be extremely challenging with lingual appliances due to their inherent strong posterior anchorage [10]. This limitation, coupled with the failure to address the protrusion, led to the exclusion of this option, as it would not meet the patient’s primary esthetic concern of reducing facial convexity.

The third option involved extracting the upper and lower right third molars to facilitate distalization of the entire upper arch and lower right quadrant, while simultaneously mesializing the lower left quadrant. This approach aimed to reduce upper incisor proclination and overjet, correct the Class II relationship on the left side, and avoid the need for an implant. This option offered a more achievable mesial movement of the lower left quadrant compared to the second option due to the coordinated distalization of the upper left quadrant.

Given the patient’s primary concern of dental protrusion, the non-extraction treatment options were declined. Consequently, the third option, involving the extraction of three third molars, was chosen to effectively reduce dental protrusion and avoid the necessity for prosthetic treatment. Regarding orthodontic appliances, the patient desired invisible options to maintain aesthetics throughout treatment. Therefore, labial fixed appliances, including ceramic brackets, were excluded because the stainless steel archwire would be visible. Clear aligners were considered as another option due to their ability to sequentially distalize the maxillary arch [11]. However, mesialization of the mandibular left quadrant would be less feasible with clear aligners due to their poor tip control during molar protraction [8]. A comprehensive orthodontic strategy incorporating lingual appliances and miniscrews was implemented to precisely control tooth movement and achieve optimal treatment outcomes.

### Treatment progress

To initiate orthodontic treatment, a 0.018 × 0.025-inch pre-adjusted self-ligating lingual appliance (LinPass, STS, Argentina) was bonded to all teeth. Bracket positioning based on a straight wire concept and three-dimensionally printed indirect bonding trays [12, 13]. A systematic approach was followed to align and level the teeth, beginning with round nickel-titanium continuous archwires and progressing through a sequence of sizes including 0.014, 0.016, and 0.016 × 0.022 inch. Approximately one month into treatment, all third molars, except for the lower left third molar, were extracted under local anesthesia.

**Fig. 3:**
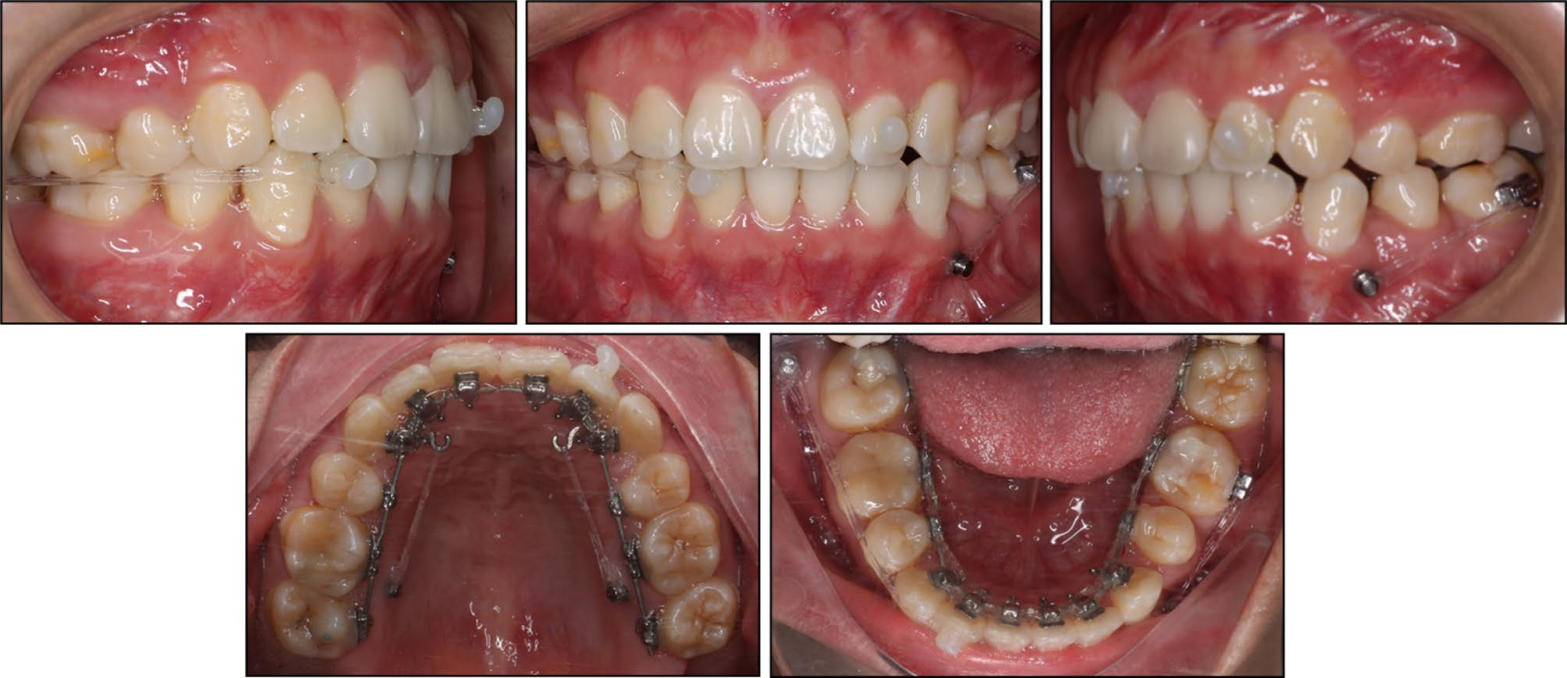
Distalization of the upper arch and the lower right quadrant and mesialization of the lower left quadrant with miniscrews

After five months of initial leveling and alignment, 0.016 × 0.022-inch stainless steel archwires were employed for both the maxillary and mandibular arches to complete arch leveling and torque expression. Three 2.0 mm diameter, 12 mm long miniscrews (Hifix, Medico, Korea), were inserted into the palatal alveolar bone between the maxillary first and second molars and the right mandibular buccal shelf. These miniscrews served as anchorage points for applying distalizing forces to the entire upper arch and the lower right quadrant. This would facilitate the correction of incisor labial inclination, excessive overjet, and the Class II dental relationship on the left side while maintaining the Class I relationship on the right side. Furthermore, a 1.6 mm diameter, 10 mm long miniscrew was inserted into the buccal alveolar bone between the lower left canine and second premolar. This miniscrew provided additional anchorage to mesialize the lower left premolar and molars (Fig. 3). In conjunction with the miniscrew anchorage, Class II elastics were employed on the left side to facilitate the correction of the full-cusp Class II relationship.

After approximately 10 months of space closure and anteroposterior correction, all the extraction spaces were closed. The patient expressed satisfaction with the improved incisor inclination and reduced lip protrusion. The final finishing stage focused on refining the tooth alignment and settling the occlusion. Aesthetic buttons were bonded to the labial surfaces of the posterior teeth for vertical elastic application to fine-tune the tooth interdigitation. Additionally, to address the lingually positioned lower right posterior segment, a consequence of distalization, cross elastics were applied from the lingual side of the lower right posterior teeth to the labial side of the upper right posterior teeth, ensuring optimal arch coordination.

After a total of 20 months, the desired tooth alignment and occlusion were achieved. The lingual appliances were debonded followed by carefully removing residual orthodontic adhesive using silicone polishers (One Gloss, Shofu, Japan) without damaging tooth enamel [14].

To ensure the long-term stability of the orthodontic treatment results and prevent relapse, a comprehensive retention protocol was implemented. Fixed retainers were bonded to both the maxillary and mandibular arches to maintain the newly achieved tooth positions. Additionally, removable Essix retainers were provided for nighttime wear, offering an extra layer of stability and preventing tooth movement during the post-treatment phase. Regular follow-up visits every 6 months were scheduled to monitor the retention progress and detect any early signs of relapse.

### Treatment results

**Fig. 4:**
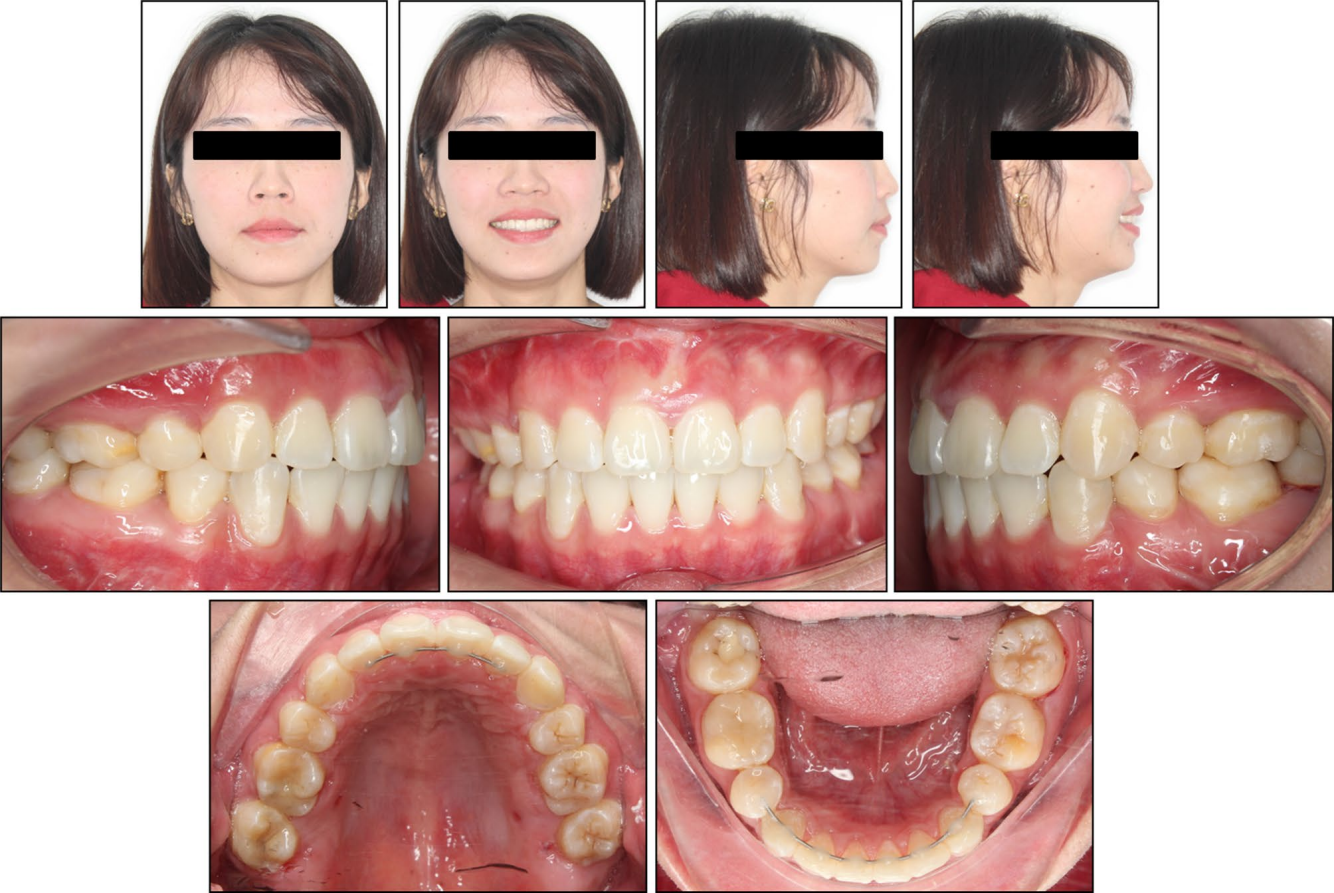
Post-treatment extraoral and intraoral photographs

The orthodontic treatment resulted in a significant improvement in the patient’s overall appearance and occlusal function. The patient’s primary concerns, including residual extraction spaces and dentoalveolar protrusion, were effectively addressed (Fig. 4).

The treatment successfully corrected the full-cusp Class II relationship on the left side to a solid Class I through a combination of lower left quadrant mesialization and upper left quadrant distalization. The existing Class I relationship on the right side was maintained with concurrent upper and lower right quadrant distalization. Ultimately, well-aligned dental arches with a leveled curve of Spee, normal overbite and overjet, and corrected upper and lower dental midlines were achieved.

**Fig. 5:**
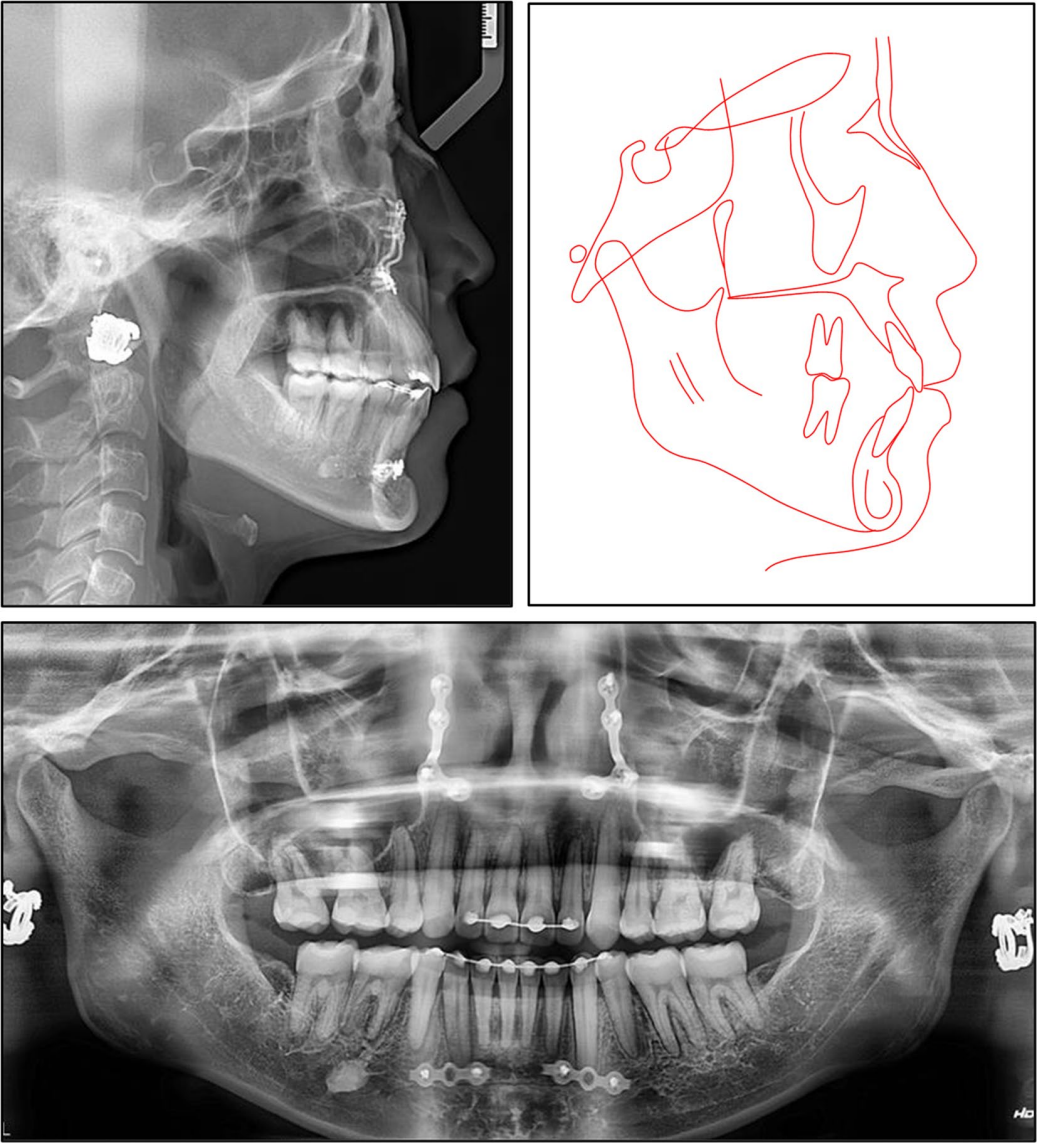
Post-treatment cephalometric and panoramic radiographs and tracing

Post-treatment cephalometric analysis revealed a slight improvement in the hyperdivergent facial pattern with the FMA angle decreasing to 30.66°, and a resulting minor improvement in the anteroposterior relationship with the ANB angle reducing to 3.10°. Upper and lower incisor inclinations also improved, with the U1-SN angle reducing to 98.85° and the L1-MP angle to 94.07°. Additionally, the soft tissue profile showed significant improvement, with the upper and lower lip positions relative to the E-line reducing to 0.01 mm and 0.51 mm, respectively. The panoramic radiograph confirmed adequate root parallelism, well-maintained alveolar bone level, and the absence of root resorption (Fig. 5).

**Fig. 6:**
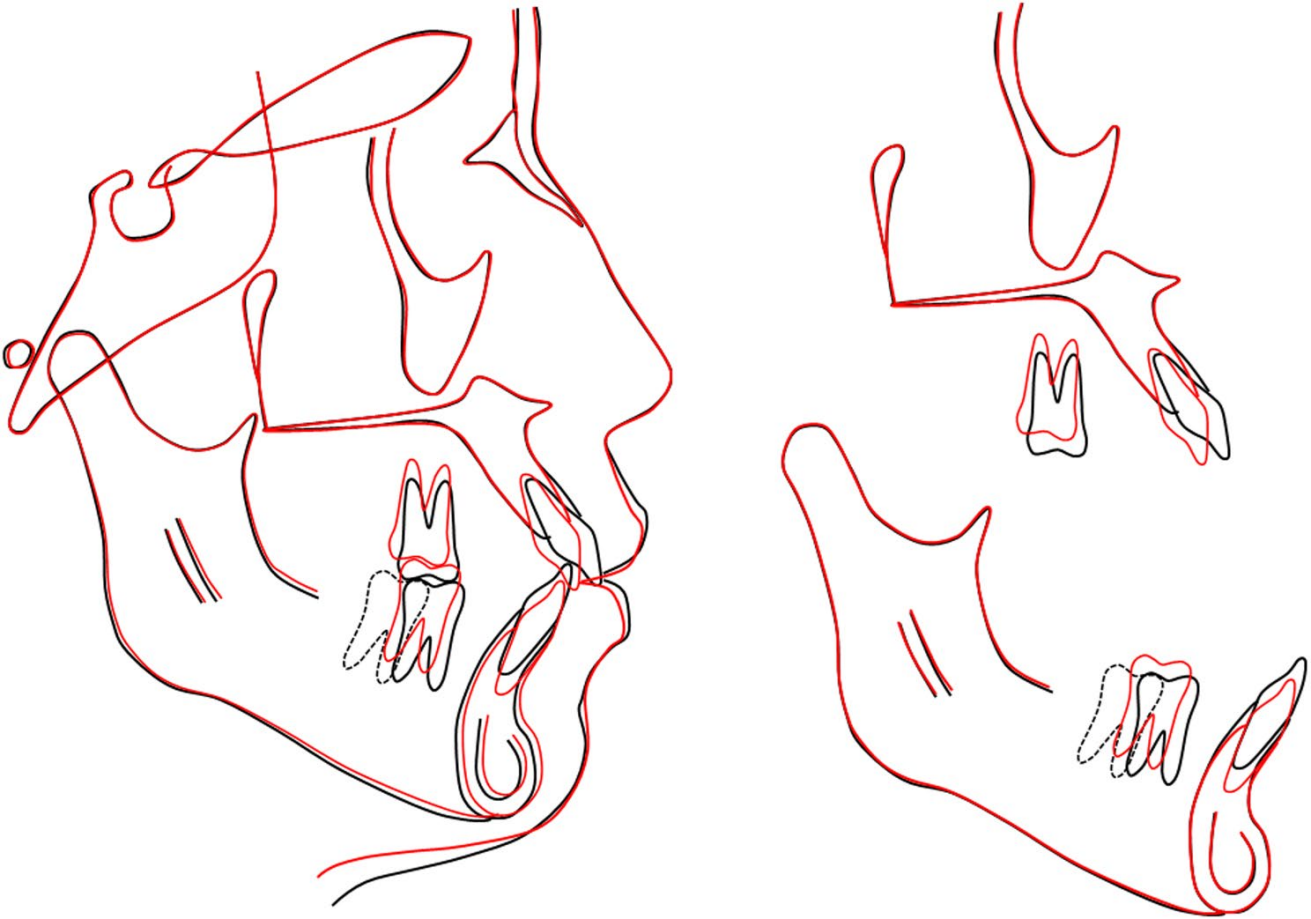
Overall, maxillary, and mandibular cephalometric superimpositions: black, pre-treatment; red, post-treatment; black dashed line, lower left second molar

Cephalometric superimpositions illustrated controlled tipping of the upper incisors, distalization and intrusion of the upper molars, and leveling of the lower curve of Spee through a combination of molar extrusion and incisor intrusion. Additionally, mesialization of the lower left molars and distalization of the lower right molars were observed, along with slight mandible counterclockwise autorotation, lip retraction, and chin point advancement (Fig. 6).

**Fig. 7:**
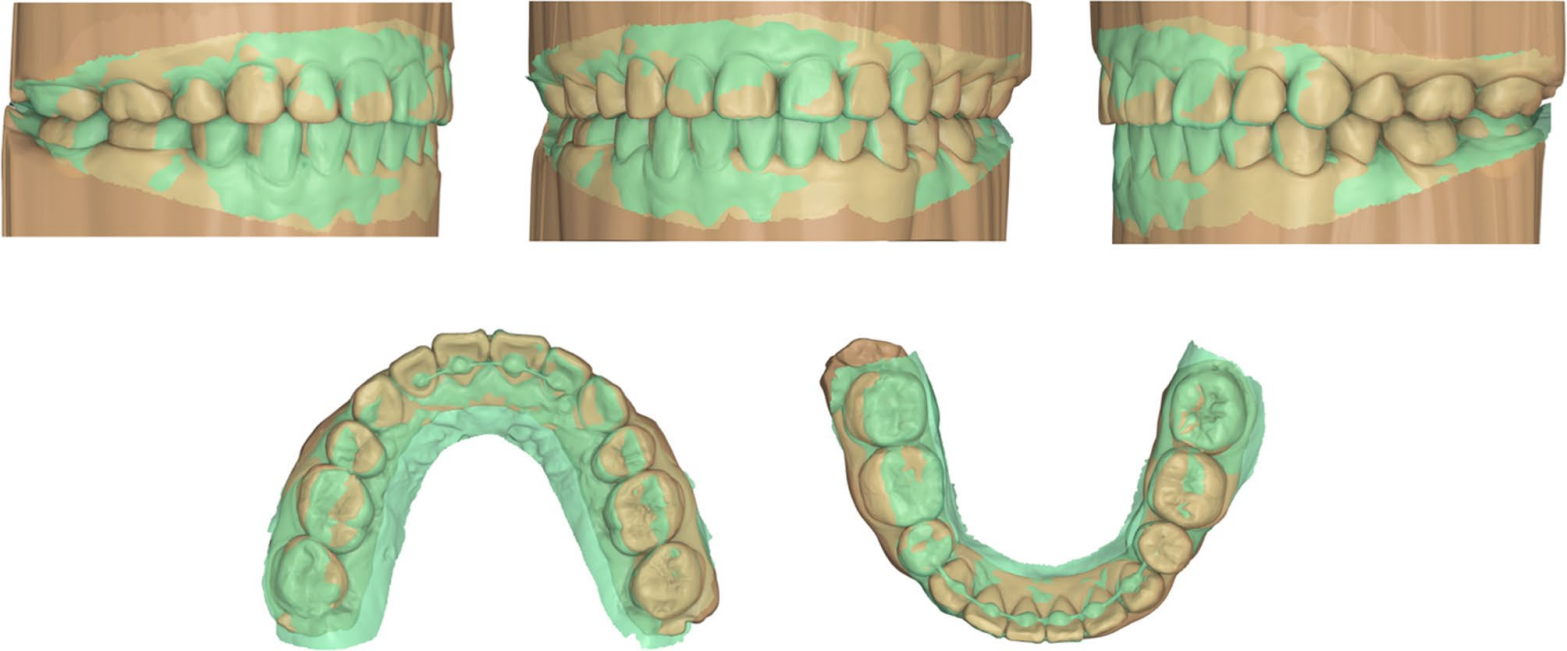
Comparison between the achieved (green) and the planned (yellow) treatment results

**Fig. 8:**
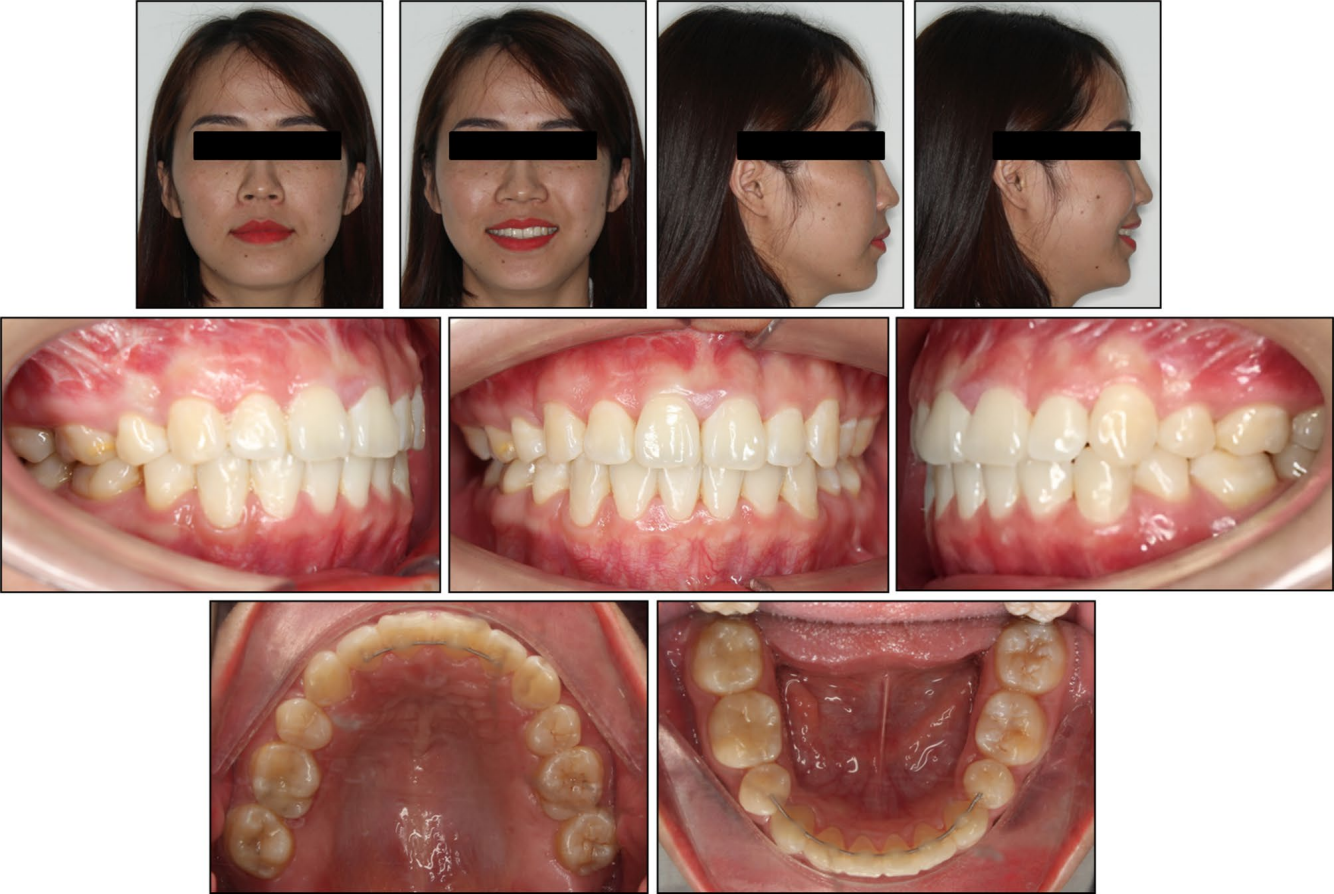
Post-retention extraoral and intraoral photographs

The patient’s satisfaction with the invisibility of the lingual orthodontic appliances highlights the aesthetic advantages of this treatment approach. The comparison between the achieved results and the planned orthodontic setup demonstrated a high agreement (Fig. 7). The 12-month post-retention evaluation revealed no signs of relapse, indicating the successful long-term retention of the correction (Fig. 8).

## Discussion

In this case, the lack of a comprehensive orthodontic and surgical plan before the initial orthognathic surgery led to a complex post-surgical occlusion, including an unresolved full-cusp Class II malocclusion on the left side. A more effective presurgical orthodontic approach could have involved extracting only the right first premolar, potentially resulting in a Class I canine relationship bilaterally and a more favorable post-surgical orthodontic phase. This would also have likely avoided the need for mandibular anterior segment osteotomy, reducing associated costs and invasiveness. The inadequate surgical plan ultimately necessitated a more complex and prolonged orthodontic treatment involving intricate biomechanics to address the residual malocclusion.

The decision to extract third molars was critical in this case, as four first premolars had already been removed during previous orthognathic surgery. This extraction strategy was supported by the patient’s hyperdivergent facial pattern, as non-extraction treatment often exacerbates vertical dimensions in such cases [15, 16]. Extracting the third molars facilitated distalization of the upper arch with miniscrews, enabling upper molar intrusion and mandibular counterclockwise autorotation, as confirmed by cephalometric superimposition. This ultimately contributed to improving the hyperdivergent pattern.

Additionally, a treatment plan without third molar extraction would have necessitated significant mesialization of the lower left molars to correct the full-cusp Class II malocclusion because the upper left molars were not distalized. This would not only be extremely challenging with lingual appliances but also pose a risk of reduced periodontal support and gingival recession of the second molar [17]. This is because the second molar would be forced to move into the narrower alveolar ridge previously occupied by the second premolar. Furthermore, the position of the lower right third molars was close to the ascending ramus, which would have prevented the distalization of the lower right quadrant to facilitate correction of the left-deviated lower dental midline without extraction.

**Fig. 9:**
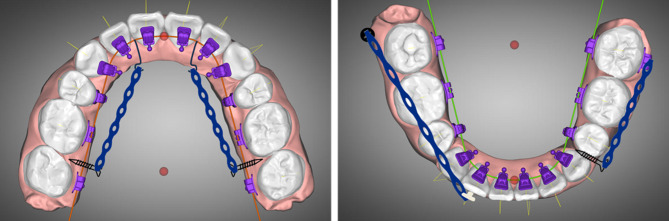
Overview of treatment mechanics using miniscrews for distalization of the upper arch and simultaneous distalization of the right side and mesialization of the left side of the lower arch

The position of the upper dental midline and facial convexity influence the biomechanical strategy for correcting Class II subdivision. In a case report by Albertini et al., the upper midline deviated towards the Class I side, and the profile was flat [6]. Consequently, distalization of the upper arch was required only on the Class II side, combined with Class II elastics to mesialize the lower arch on the same side. However, this case presented a centered upper midline and dentoalveolar protrusion, necessitating distalization of the entire upper arch and the lower arch on the Class I side, while mesializing the lower arch on the Class II side (Fig. 9).

In this case, various treatment alternatives were considered, including clear aligners and traditional fixed appliances. Clear aligners offer the advantage of being aesthetic and comfortable for patients who are compliant. However, for complex malocclusions like the one in this case, clear aligners presented significant challenges. One major drawback is the dependence on patient compliance, as clear aligners require regular removal and re-insertion, which can be inconvenient when applying forces through miniscrews for mesializing and distalizing movements. Each time the aligner is removed, there is potential for disruption in the force application, which can hinder effective tooth movement. Additionally, aligners may not provide adequate control over molar movement during protraction and may lead to less predictable results when extensive tooth movements are required. In contrast, the fixed lingual appliances used in this case, supported by miniscrews, provided more predictable and precise control over tooth movement. These appliances allowed for the application of more consistent and controlled forces, addressing the patient’s complex skeletal and dental discrepancies efficiently.

The unique biomechanics of lingual orthodontics present challenges in molar mesialization. Protraction forces applied to posterior teeth with lingual appliances generate buccal root torque and distopalatal rotation of the molars, increasing cortical bone anchorage, particularly for mandibular molars [10]. This effect is exacerbated when the mesial alveolar bone is atrophic due to a missing tooth, making molar protraction more difficult. However, intentional protraction of mandibular molars with miniscrew anchorage offers several advantages in these cases. Miniscrews provide absolute anchorage, allowing for molar movement without reciprocal forces on anterior teeth. Additionally, buccally placed miniscrews counteract the distopalatal rotation caused by lingual protraction, reducing cortical bone anchorage and facilitating more efficient mesialization. However, the buccal retraction force from the lower right miniscrew exacerbated distopalatal rotation, tending to move the lower right molars lingually and creating lower arch constriction relative to the upper arch. To counteract this and coordinate the arches, cross elastics were necessary. The direction of these elastics also aided in correcting the lower midline deviation.

In recent years, the integration of virtual orthodontic setups has allowed orthodontists to visualize and predict potential treatment outcomes with greater precision. By leveraging these tools, accurate bracket placements and customized straight lingual archwire templates can be generated. These ideal bracket positions can be transferred to the patient’s dentition with directly printed indirect bonding trays, resulting in tooth alignment that closely matches the planned orthodontic outcome [12, 18]. The overlay of the achieved post-treatment results and the planned ones revealed a remarkable congruence (Fig. 7), indicating the high accuracy and predictability of digital workflows.

The role of the digitally planned lingual appliances in this case is crucial. Digital planning enhanced the accuracy of bracket positioning, ensuring precise alignment from the start of treatment. This increased accuracy not only improved the predictability of tooth movement but also reduced treatment time by streamlining the initial alignment process. With digital tools, customized archwire designs and individualized bracket placements were made possible, which contributed to both faster and more efficient treatment. Additionally, digital planning has been shown to enhance patient satisfaction, as the treatment process becomes more predictable, and patients can visualize their treatment outcomes through advanced digital simulations.

This case highlights a relatively straightforward re-treatment; however, long-term follow-up is essential to assess stability and the overall outcome. A 1-year post-treatment evaluation, as conducted in this case, is reasonable and provides valuable early insight into treatment success. Studies have shown that while significant relapse mostly occurs in the short term, occlusal characteristics and maxillary anterior crowding tend to stabilize from the short-term to the long-term post-retention stages [19, 20]. Therefore, the 1-year follow-up in this case serves as an initial check for stability, but extended observation beyond this period is crucial to fully assess the effectiveness of the intervention and detect any potential relapse.

## Conclusions

This case report highlights the successful management of a complex Class II subdivision malocclusion arising from previous suboptimal orthognathic surgery. The integration of digital planning and miniscrew-assisted anchorage was critical to achieving the desired results. This approach not only allowed for precise control over tooth movement but also minimized aesthetic concerns, thanks to the use of lingual appliances. For clinicians, this case emphasizes the importance of combining advanced digital tools with effective anchorage systems to handle challenging malocclusions. Additionally, it highlights the necessity of addressing both functional and aesthetic outcomes when treating adult patients, especially those with a history of failed orthognathic surgery. The case also reinforces the potential of digital workflows and miniscrew-assisted orthodontics to enhance precision and predictability in complex malocclusions.

## Data Availability

All data generated or analyzed during this study are included in this published article.

## Abbreviations

SNA: Sella nasion point A
SNB: Sella nasion point B
ANB: Point A nasion point B
FMA: Frankfort mandibular plane angle
U1: Upper central incisor
L1: Lower central incisor
NB: Nasion point B
NA: Nasion point A
MP: Mandibular plane
